# Analgesic Effect of 17β-Estradiol on Nucleus Paragigantocellularis Lateralis of Male Rats Mediated Via GABA_A_ Receptors

**DOI:** 10.15412/J.BCN.03080107

**Published:** 2017-01

**Authors:** Roghaieh Khakpay, Maryam Azaddar, Fatemeh Khakpay, Homeira Hatami Nemati

**Affiliations:** 1. Department of Animal Science, Faculty of Natural Sciences, University of Tabriz, Tabriz, Iran.; 2. Department of Biology, Faculty of Basics Sciences, Varamin Branch, Islamic Azad University, Pishva, Iran.

**Keywords:** 17β-Estradiol, GABA_A_ receptor, Pain, Analgesia

## Abstract

**Introduction:**

Beside its autonomic functions, the nucleus paragigantocellularis lateralis (LPGi) is involved in the descending pain modulation. 17β-Estradiol is a neuroactive steroid found in several brain areas such as LPGi. Intra-LPGi microinjection of 17β-estradiol can elicit the analgesic responses. 17β-Estradiol modulates nociception by binding to estrogenic receptors as well as allosteric interaction with other membrane-bound receptors like GABA_A_ receptors. This study aimed to examine the role of GABA_A_ receptors in the pain modulating effect of intra-LPGi injection of 17β-estradiol.

**Methods:**

To study the antinociceptive effects of 17β-estradiol, cannulation into the LPGi nucleus of male Wistar rats was performed. About 500 nL of drug was administered 15 minutes prior to formalin injection (50 μL of 4%). Then, formalin-induced flexing and licking behaviors were recorded for 60 minutes. For evaluating the role of GABA_A_ receptors in the estradiol-induced pain modulation, 17β-estradiol was administered into the LPGi nucleus 15 minutes after the injection of 25 ng/μL bicuculline (the GABA_A_ receptor antagonist). Then, the formalin-induced responses were recorded.

**Results:**

The results of the current study showed that intra-LPGi injection of 17β-estradiol decreased the flexing duration in both phases of formalin test (P<0.001); but it only attenuated the second phase of licking behavior (P<0.001). 17β-estradiol attenuated the second phase of formalin test of both behaviors (P<0.001). Bicuculline prevented the antinociceptive effect of intra-LPGi 17β-estradiol in both first and second phases of formalin-induced responses (P<0.001).

**Conclusion:**

According to the results of this study, the analgesic effect of intra-LPGi 17β-estradiol on the formalin-induced inflammatory pain might be mediated via GABA_A_ receptors.

## 1. Introduction

The processing of the painful information is mediated by various structures of nociceptive system. The ascending pathways transfer the details of noxious stimuli from the periphery to supraspinal centers such as the nucleus paragigantocellularis (Potes, Neto, & Castro-Lopes, 2006). The nucleus paragigantocellularis is a widespread part of the reticular formation; it is divided into dorsal and lateral parts. The lateral part of the nucleus paragigantocellularis is called nucleus paragigantocellularis lateralis or LPGi. It is a reticular nucleus in the rostral medulla oblongata and involved in pain modulation (Khakpay, Barani, & Hatami Nemati, 2014) as well as autonomic functions like cardiovascular regulation (van Bockstaele, Akaoka, & Aston-Jones, 1993), control of sleep-wake cycle, respiratory system (Arita, Kogo, & Ichikawa, 1988), and sexual behavior (Fathi-Moghaddam, Kesmati, & Mohammad Pour Kargar, 2006).

Neurosteroids are steroids synthesized from cholesterol in the central nervous system (Compagnone & Mellon, 2000; Mellon & Vaudry, 2001) where they control neuronal excitability (Smith, 2003). In contrast to the genomic influences interceded by intracellular steroid receptors (McEwen, 2002; Vasudevan & Pfaff, 2008).Neurosteroids like 17β-estradiol rapidly enhance the function of GABA_A_ receptors by interaction with its membrane-bound receptors (Rupprecht & Holsboer, 1999).

17β-Estradiol as a neuroactive steroid quickly modulates the synaptic transmission and plasticity in the adult brain, even outside areas concerned with reproductive behavior (Grassia et al., 2012; Hajszan, MacLusky, & Leranth, 2008; Isgor & Sengelaub, 2003; Sakuma, 2009). In LPGi (Khakpay et al., 2014), 17β-estradiol modulates nociception by binding to its receptors as well as allosteric interaction with other membrane-bound receptors like glutamate and GABA_A_ receptors (Khakpay, Semnanian, Javan, & Janahmadi, 2010b; Potes et al., 2006).

Current studies (Hosie, Wilkins, da Silva, & Smart, 2006) have reported that α1β2δ2-containing GABA receptors have distinct steroid binding sites. There are two discrete sites; one considered to bind steroids, which reinforces chloride current of GABA_A_ receptors and the second site planned for steroid dependent activation of the receptor. Every binding site is confined in a physically isolated hydrophobic hole which can bind a single steroid molecule (Smith, 2003). The δ-subunit-containing GABA receptor subtypes are sensitized to steroid modulation (Belelli, Casula, Ling, & Lambert, 2002; Brown, Kerby, Bonnert, Whiting, & Wafford, 2002; Wohlfarth, Bianchi, & Macdonald, 2002). GABA_A_ receptors are extensively distributed in different regions of the central nervous system (Yang, Ma, Feng, Dong, & Li, 2002), including rostral ventrolateral medulla (RVLM) (Fields & Basbaum, 1999; Foley et al., 2003).

In the rat brain, the RVM includes the nucleus raphe magnus (NRM), nucleus reticularis gigantocellularis pars α and LPGi (Fields, Heinricher, & Mason, 1991; Mason, 1999; Willis Jr. & Coggeshall, 2004; Yang et al., 2002). Also, GABA ergic neurons have been identified in the LPGi (Dehkordi et al., 2007). On the other hand, GABA_A_ receptor subunit expression was largely unaltered at the chronic time points (Drexel et al., 2015; Pavlov et al., 2011).

Considering the active role of LPGi nucleus (Aston-Jones et al., 1991) and interaction of 17β-estradiol with GABA_A_ receptors in the modulation of pain (Khakpay, Semnanian, Javan, & Janahmadi, 2010b), this study was designed to assess the role of the membrane-bound GABA_A_ receptors in the pain modulating effect of intra-LPGi injection of 17β-estradiol of male rats.

## 2. Methods

### 2.1. Animals

Experiments were performed on adult male Wistar rats (weighing 200–270 g) purchased from Razi Institute (Hesarak Karj, Iran). Animals were housed at 22–24°C under 12:12 h light/dark cycle. Food and water were accessible ad libitum in their cage. The experiments were performed between 8:00 and 16:00, 5 days/week. All research and animal care procedures were performed according to international guidelines on the use of laboratory animals (NIH Publication No. 80-23, revised 1996) and were approved by Ethics Committee for Animal Research of Tabriz University.

The animals were randomly divided into 7 groups, including the control group (formalin test in the intact animals), the second group or sham (only cannulation and formalin test), the third group (intra-LPGi injection of saline and formalin test), the fourth group (intra-LPGi injection of 0.8 μmol 17β-estradiol and formalin test), the fifth group (intra-LPGi injection of 5 μmol bicucul-line and formalin test), the sixth group (intra-LPGi injection of 2.5 μmol bicuculline and formalin test), and the seventh group (intra-LPGi injection of 2.5 μmol bicuculline 15 minutes before the intra-LPGi administration of 0.8 μmol 17β-estradiol and formalin test).

### 2.2. Procedure

The animals were gently handled 15 min/d for a week before the experiment for acclimatization. On the day of the procedure, the rats were anesthetized with intraperitoneal injection of ketamine (60 mg/kg) and xylazine (7.5 mg/kg). Animals were unilaterally implanted with a guide cannula (23 gauge) - equipped with a 30-gauge stylet- into the right LPGi (coordinates from Bregma: AP: − 11.9 mm, L:±1.6 mm, DV: 10.4 mm) (Paxinos & Watson, 2004). A stainless steel screw and acrylic cement (Dentimax, The Netherlands) were used to fix the guide cannula to the skull. After 5–7 days recovery period, the formalin test was performed on all animals.

### 2.3. Injections

**Figure 1:**
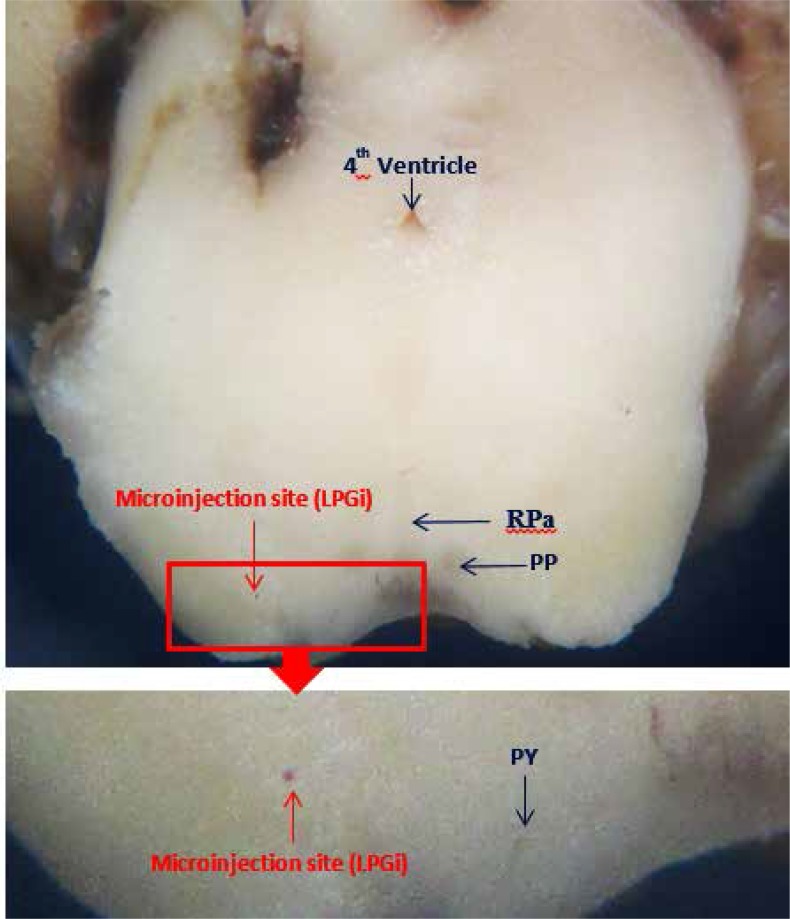
The histological landmarks and confirmation for accurate drug injections into the LPGi nucleus.

Intra-LPGi injections were done as previously described (Aloisi & Ceccarelli, 1999). Considering the contralateral ascending of the nociceptive fibers, all injections were unilaterally done into the right LPGi through the guide cannula using an injection needle (30 gauge) connected by polyethylene tubing to a 0.5-μL Hamilton microsyringe (Hamilton, Switzerland). Nociceptive fibers ascend contralaterally to the PGi, including LPGi nucleus. The injection needle was replaced by the stylet with its tip 2 mm beyond the guide cannula. All substances were injected in a volume of 500 nL. The needle was removed and the stylet replaced 60 seconds after infusing the chemical substance (Figure 1).

### 2.4. Formalin test

Animals were adapted to the experimental room and test chamber for 20 min/d, for 2 days before the experiment. In order to study the involvement of the GABA_A_ receptors in the antinociceptive effect of 17β-estradiol, bicuculline were injected 15 minutes prior to 17β-estradiol administration, and then formalin test (Dubuisson & Dennis, 1978) was done 15 minutes after 17-estradiol injection. Therefore, 50 μL of 4% formalin was subcutaneously injected into the rats’ left hindpaws using a 30-gauge needle (Khakpay et al., 2014). Following the formalin injection, the animal was returned to the test chamber (a square transparent plexiglas cage, 30×30×30 cm) and the duration of hindpaw flexing and licking responses were observed for 60 minutes (Aloisi, Ceccarelli, & Lupo, 1998; Khakpay et al., 2014; Khakpay et al., 2010b; Wheeler-Aceto & Cowan, 1991).

The data collected between 0–7 minutes after formalin injection were considered as the first phase or acute phase and the data collected 15–60 minutes after formalin injection were considered as the second phase or chronic phase (Khakpay et al., 2014; Mahmoudi & Zarrindast, 2002). After the experiment, the rats were killed by diethyl ether and their brains were removed and checked for the correct cannula placement in the LPGi (Figure 1). Only data from animals with correct placement of cannula were included in the analysis.

### 2.5. Statistical analysis

All data were calculated using SPSS and presented as mean±S.E.M. One-way analysis of variance (ANOVA) with post hoc Tukey test were used to analysis of differences between groups. P<0.05 was considered to be statistically significant.

## 3. Results

There were no statistically significant differences between sham operated (LPGi cannulation without intra-LPGi injections), saline (intra-LPGi injections of saline) and control (intact animals) groups; therefore they were excluded in the result section. The mean response between 0 to 7 minutes after formalin injection reflects the acute phase and the mean response between 15 and 60 minutes reflects the chronic phase.

### 3.1. Effect of 17β-estradiol on formalin-induced responses

**Figure 2:**
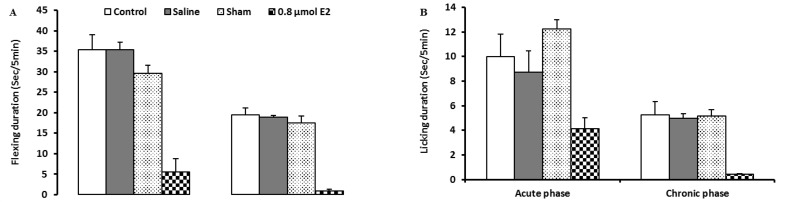
Effect of intra-LPGi injection of 0.8 μmol 17β-estradiol on flexing (A) and licking (B) behaviors following injection of 50 μL of 4% formalin into the plantar surface of the left hindpaw, the graph shows data for the acute and the chronic phase of formalin-induced responses in comparison with control, sham, and saline-injected animals. The nociceptive responses are presented by mean ± SEM of flexing and licking duration of 6 rats per group. *Indicates significant difference from control group (P<0.05), ***Indicates significant difference from control group (P<0.001).

Intra-LPGi injections of 0.8 μmol 17β-estradiol significantly reduced flexing duration both the acute and chronic phases (P<0.001, Figure 2A).

Intra-LPGi injections of 0.8 μmol of 17β-estradiol significantly reduced licking duration just in the chronic phase (P<0.001, Figure 2B).

To clarify the mechanism of the antinociceptive effect of 17β-estradiol and the involved receptors, we tried to find a suitable dose of GABA_A_ antagonists without any significant effect on nociception. These experiments were performed by bicuculline.

### 3.2. Effects of bicuculline on formalin-induced responses

**Figure 3:**
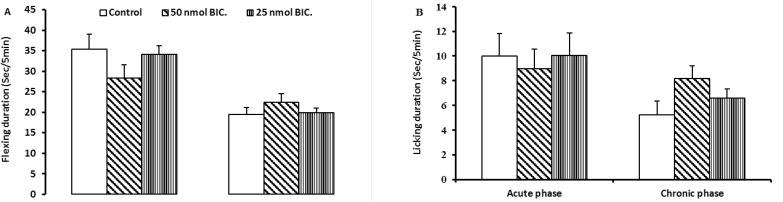
Nociceptive responses (flexing A and licking B) during the acute and the chronic phases of the formalin test in rats treated with 25 and 50 nmol bicuculline 15 minutes before formalin injection (4%, 50 μL). The data are represented as mean±SEM for six rats. *Indicates significant difference from control group (P<0.05).

Intra-LPGi injections of 25 and 50 nmol of bicuculline did not show any significant differences with the control group (Figure 3A) i.e. bicuculline had no pronociceptive effect and interference with analgesic effect of 17β-estradiol.

Intra-LPGi injection of 50 nmol of bicuculline significantly increased licking response of rats in chronic phase (P<0.05, Figure 3B). The licking response in both phases of formalin pain was not significantly affected by intra-LPGi administration of 25 nmol bicuculline (Figure 2B). Then, 25 nmol of bicuculline was considered as the proper dose for the rest of experiments.

For studying the possible involvement of membrane-bound GABA_A_ receptors in the antinociceptive effect of 17β-estradiol, bicuculline were applied 15 minutes before the injection of 17β-estradiol and pain-related behaviors were examined following formalin injection.

### 3.3. Effects of GABA_A_ receptor antagonists on the antinociceptive effect of 17β-estradiol

**Figure 4:**
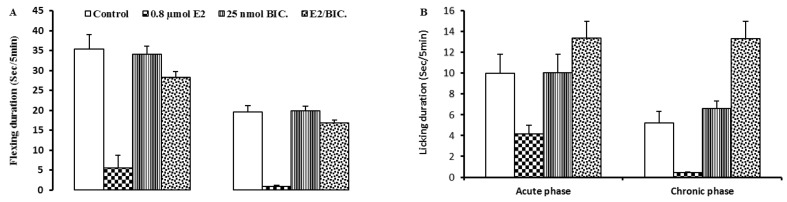
Effect of pretreatment with GABA_A_ receptor antagonists on the antinociceptive effect of intra-LPGi 17β-estradiol on the flexing and the licking responses, bicuculline (25 nmol) was administered 15 minutes before intra-LPGi injection of 0.8 μmol 17β-estradiol and formalin test was done 15 minutes after 17β-estradiol injection (E2/Bic. group). Data are presented as mean±SEM for 6 rats and significant differences between the 17β-estradiol and the antagonists groups are shown by *** which represents (P<0.001) compared to 17β-estradiol group.

Pretreatment of LPGi nucleus with 25 nmol bicuculline 15 minutes before 17β-estradiol injection significantly reversed the analgesic effect of 0.8 nmol intra-LPGi 17β-estradiol on the flexing duration in both acute and chronic phases of formalin-induced pain (P<0.001, Figure 4A). Bicuculline administration into the LPGi nucleus 15 minutes after 17β-estradiol injection has moderate to strong antagonistic effect on the flexing behavior in both acute and chronic phases of formalin test and reversed it approximately to the control level.

Application of 25 nmol bicuculline 15 minutes before intra-LPGi 17β-estradiol administration significantly prevented the antinociceptive effect of 17β-estradiol on licking behavior in the acute phase as well as chronic phase of formalin test (P<0.001, Figure 4B). Bicuculline has very potent antagonistic effect on the licking behavior in both acute and chronic phases of formalin test and reversed it to the control level (Figure 4B).

## 4. Discussion

Our results indicated that 17β-estradiol treatment of LPGi nucleus attenuated the chronic phase of lickingbehavior. Also, 17β-estradiol decreased the flexing duration in the both phases of formalin test. GABA_A_ receptor antagonist, bicuculline, completely reversed the attenuation of 17β-estradiol-induced flexing and licking behaviors. Since the analgesic effect of 17β-estradiol was entirely eliminated by bicuculline pretreatment, the antinociceptive effect of intra-LPGi injection of 17β-estradiol is possibly mediated by the membrane-bound GABA_A_ receptors.

Several neuronal activities of brain are controlled by steroid hormones through changing the receptive field area and the neuronal communications in many brain regions (Khakpay et al., 2010a). For surveying the centrally mediated influences of 17β-estradiol on the formalin-induced persistent pain, intra-LPGi injection of 17β-estradiol was done in the male rats.

17β-Estradiol as a neurosteroid enhances the function of GABA_A_ receptors by binding directly to the receptors in the cell membrane (Rupprecht & Holsboer, 1999). Our previous study showed that a part of this analgesic effect in the formalin-induced inflammatory pain is mediated through intracellular estrogen receptors (Khakpay et al., 2014; Khakpay, Barani, & Hatami Nemati, 2015); our results in the current study indicated that the other part of this effect is possibly mediated through allosteric interactions and or its direct bind to the membrane-bound GABA_A_ receptors.

Most of the pain literature has mentioned the role of the sex steroids in the behavioral responses to acute nociceptive stimuli, but the results have been contradictory (Felton & Auerbach, 2004; Gordon & Soliman, 1996; Madeira & Lieberman, 1995). Particularly, estradiol has been reported to change (increase in some studies and decrease in other studies) the threshold of responses to the hot plate and latencies in the tail flick assays (Khakpay et al., 2010a; Stoffel, Ulibarri, & Craft, 2003; Stoffel, Ulibarri, Folk, Rice, & Craft, 2005). In this study, the formalin test, a common model for studying both acute and persistent pain, was used to investigate the possible analgesic effect of 17β-estradiol and its underlying mechanisms in LPGi nucleus.

In the present study, intra-LPGi administration of 0.8 μmol of 17β-estradiol had a significant antinociceptive effect on the first and the second phases of formalin-induced flexing behavior. Similarly, intra-LPGi 17β-estradiol had a significant pain relieving effect only on the second phase of formalin-induced licking behavior. It can be hypothesized that intra-LPGi administration of 17β-estradiol affects either estrogen receptors or the membrane-bound GABA_A_ receptors of LPGi which are able to modulate pain-evoked neural activity in the spinal and supraspinal circuits. Therefore, the attenuation of the first and second phases of the formalin-induced responses confirms that estradiol treatment of LPGi affects the nociceptive inputs as well as their processing in the LPGi nucleus.

Neurosteroids and protein kinases are among the most potent modulators of the GABA_A_ receptor. When they act individually, they can enhance or depress receptor functions depending on the nature of the neurosteroid, protein kinase, and the subunit combination of the receptors (Adams, Thomas, & Smart, 2015; Belelli & Lambert, 2005). Furthermore, the positive allosteric modulators (PAMs) of GABA_A_ receptor mediate robust analgesia in the spinal cord after the injury (Munro, Erichsen, Rae, & Mirza, 2011).

This study was designed for assessing the involvement of GABA_A_ receptor in the pain modulatory influences of 17β-estradiol. Therefore, bicuculline, a specific antagonist of the heterodimeric GABA_A_ receptor (Khakpay et al., 2010a; Pathirathna et al., 2005; Sahebgharani, Hossein-Abad, & Zarrindast, 2006) was selected. To this end, we tried to find a suitable dose of antagonist without any significant effect on nociception. In the present study, 50 nmol intra-LPGi administration of bicuculline showed a mild pronociceptive effect, but the dose of 25 nmol did not show any significant nociceptive response. Therefore, the lower dose of bicuculline was chosen because it could not interfere with analgesic effect of 17β-estradiol.

There is evidence that bicuculline microinjection into the LPGi nucleus has either analgesic (Kaneko & Hammond, 1997; Loomis, Khandwala, Osmond, & Hefferan, 2001) or hyperalgesic (Sahebgharani et al., 2006; Saleh & Saleh, 2001) effects depending on the dose. In the current study, intra-LPGi injection of higher dose (50 nmol) of bicuculline increased only the second phase of the licking response. Consistent with our findings, Dirig and Yaksh reported that intrathecal injection of muscimol, a GABA_A_ agonist, blocked painful behavior evoked by intraplantar injection of formalin (Dirig & Yaksh, 1995). Also, Kaneko and Hammond indicated that intrathecal injection of bicuculline significantly increased the number of flinches and weighted nociceptive behavior scores in the second phase in rats (Kaneko & Hammond, 1997). Similar to our findings, Lee et al. showed that microinjection of muscimol (1 or 2 μg/0.5 μL) into the medial septum of awake rats suppressed both licking and flinching behaviors during the formalin test of inflammatory pain (Lee et al., 2011). Furthermore, intrathecal application of bicuculline, or the glycine receptor antagonist, strychnine, can elicit allodynia (Torsney & Mac-Dermott, 2006).

In our study, pretreatment with bicuculline reversed the 17β-estradiol-induced decrement in both flexing and licking behaviors in the first phase as well as the second phase of formalin test. Our results showed that a part of the analgesic effect of intra-LPGi 17β-estradiol on the formalin-induced inflammatory pain is probably mediated by GABA_A_ receptors. Consistent with our results, McGowan et al. indicated that antinociception produced by activation of neurons in the nucleus reticularis gigantocellularis pars α is partly mediated by the action of GABA_A_ receptors in the spinal cord (McGowan & Hammond, 1993). Pretreatment of LPGi nucleus with bicuculline significantly reversed both acute and chronic phases of the flexing as well as licking behaviors. Mahmoudi and Zarrindast showed that intracerebroventricular injection of different doses of muscimol, a GABA_A_ agonist, dose-dependently decreases both phases of formalin-induced pain behavior. The muscimol-induced responses in both phases of formalin test reduced by bicuculline (Mahmoudi & Zarrindast, 2002).

Similar to our results, they concluded that the stimulation of GABA_A_ receptors is responsible for antinociception in the formalin test (Mahmoudi & Zarrindast, 2002). In agreement with our results, Suzukia et al. reported that inhibition of formalin-induced nociceptive behavior is mediated by activation of GABA_A_ receptors in the spinal cord (Suzuki, Yuzurihara, Hibino, Yano, & Kase, 2009). Bicuculline increases the evoked thalamic response in males and ovariectomized female rats. Thus, estrogen inhibits neurotransmission in the PBN via interaction with the GABA receptor to modulate the flow of visceral pain to the thalamus (Saleh & Saleh, 2001).

In conclusion, our data suggest that 17β-estradiol-induced analgesia in the LPGi nucleus is possibly mediated by non-estrogen receptors. With regard to the membrane-bound receptors, GABA_A_ receptors seems to be involved in 17β-estradiol-mediated antinociception in the LPGi; however, this topic needs more investigation through molecular and electrophysiological approaches.
